# Wnt7b overexpression in osteoblasts stimulates bone formation and reduces obesity in mice on a high-fat diet

**DOI:** 10.1093/jbmrpl/ziae122

**Published:** 2024-09-23

**Authors:** Fangfang Song, Tyler Marmo, Chao Song, Xueyang Liao, Fanxin Long

**Affiliations:** State Key Laboratory of Oral & Maxillofacial Reconstruction and Regeneration, Key Laboratory of Oral Biomedicine Ministry of Education, Hubei Key Laboratory of Stomatology, School & Hospital of Stomatology, Wuhan University, Wuhan, Hubei 430079, China; Translational Research Program in Pediatric Orthopedics, Department of Surgery, The Children’s Hospital of Philadelphia, Philadelphia, PA 19104, United States; Translational Research Program in Pediatric Orthopedics, Department of Surgery, The Children’s Hospital of Philadelphia, Philadelphia, PA 19104, United States; Translational Research Program in Pediatric Orthopedics, Department of Surgery, The Children’s Hospital of Philadelphia, Philadelphia, PA 19104, United States; Translational Research Program in Pediatric Orthopedics, Department of Surgery, The Children’s Hospital of Philadelphia, Philadelphia, PA 19104, United States; Translational Research Program in Pediatric Orthopedics, Department of Surgery, The Children’s Hospital of Philadelphia, Philadelphia, PA 19104, United States

**Keywords:** Wnt7b, obesity, osteocalcin, high-fat diet (HFD), osteoblast

## Abstract

Previous studies have shown that Wnt7b potently stimulates bone formation by promoting osteoblast differentiation and activity. As high-fat feeding leads to obesity and systemic metabolic dysregulation, here we investigate the potential benefit of Wnt7b overexpression in osteoblasts on both bone and whole-body metabolism in mice fed with a high-fat diet (HFD). Wnt7b overexpression elicited massive overgrowth of trabecular and cortical bone but seemed to ameliorate body fat accumulation in mice with prolonged HFD feeding. In addition, Wnt7b overexpression modestly improved glucose tolerance in male mice on HFD. Collectively, the results indicate that targeted overexpression of Wnt7b in osteoblasts not only stimulates bone formation but also improves certain aspects of global metabolism in overnourished mice.

## Introduction

The skeleton is increasingly recognized for secreting endocrine factors to regulate the function of extra-skeletal tissues. Most notably, Fgf23 secretion by osteocytes is critical for maintaining the homeostasis of serum phosphate.^1^ In addition, osteocalcin, the most abundant non-collagen protein produced by osteoblasts, has been implicated in the regulation of global glucose metabolism.^2–4^ In particular, the uncarboxylated form of osteocalcin released into the circulation upon bone resorption has been proposed to increase insulin secretion via direct regulation of pancreatic beta cells.^2^ However, such an endocrine role has been challenged by more recent work where additional osteocalcin knockout mouse strains exhibited no metabolic anomaly.^5^^,^^6^ Another study with osteocalcin-null rats also questioned the effect of osteocalcin on systemic glucose homeostasis.^7^ Nonetheless, administration of recombinant osteocalcin has been shown to improve glucose metabolism and alleviate obesity in mice caused by a high-fat diet (HFD) or chemical-induced hyperphagia.^8^^,^^9^ Thus, irrespective of the role of endogenous osteocalcin, pharmacological levels of osteocalcin may improve systemic glucose metabolism, but this proposition needs to be further tested.

Wnt (Wingless/int-1) proteins, a family of secreted glycoproteins, are well established to promote bone mass accrual in both mice and humans. In particular, loss-of-function mutations in the WNT  co-receptor LRP5 (low-density lipoprotein receptor-related protein 5) result in low bone mass in osteoporosis-pseudoglioma syndrome, whereas gain-of-function mutations in LRP5 markedly increase bone mass in otherwise healthy individuals.^10^^,^^11^ Furthermore, loss-of-function or loss-of-expression mutations of SOST (sclerostin), a secreted antagonist that prevents WNTs from binding to LRP5, cause high bone mass in sclerosteosis or Van Buchem disease, respectively.^12^^,^^13^ Mice engineered to express disease-relevant Lrp5 or Sost mutations recapitulate the corresponding human bone phenotypes.^14–16^ Interestingly, studies of the Lrp5-null mice also uncovered defects in glucose and cholesterol metabolism besides the expected osteopenia.^17^ Conversely, Sost-null mice exhibited improved insulin sensitivity and reduced adiposity, concurrent with osteosclerosis.^18^ However, neither study examined the potential contribution of osteocalcin to the metabolic phenotypes. More recently, mice expressing a high bone mass Lrp5 mutation not only prevented diabetic osteopenia but also improved peripheral glucose metabolism in a type I diabetes mouse model.^19^ Here, the metabolic improvement was likely independent of osteocalcin as its circulating levels were unaltered by the Lrp5 mutation in the diabetic mice.^19^ Overall, the studies to date support concordant regulation of bone accrual and glucose metabolism in mice with altered Wnt signaling, but the global genetic modifications in those studies preclude clear delineation of a bone-derived endocrine function.

We have previously demonstrated a potent bone anabolic function for Wnt7b. Wnt7b is normally expressed by the osteogenic perichondrium during long bone development, and its deletion resulted in a temporary delay in ossification during embryogenesis.^20^^,^^21^ Postnatal overexpression of Wnt7b specifically in osteoblasts greatly stimulated bone formation in young, aged, or glucocorticoid-treated mice.^22–24^ The increased bone accrual was associated with notable increases in the circulating levels of both carboxylated and uncarboxylated osteocalcin.^24^ Furthermore, bone anabolic Wnt signaling has been shown to stimulate glucose, glutamine, and fatty acid metabolism in osteoblasts.^25–28^ Therefore, the Wnt7b-overexpressing mice provide a useful model for determining the potential metabolic benefit of increased bone anabolism.

Here we report that targeted overexpression of Wnt7b in osteoblasts markedly stimulated bone formation in mice fed an HFD. Despite causing a 3-fold increase in circulating uncarboxylated osteocalcin, Wnt7b overexpression did not improve fasting blood glucose or insulin sensitivity in the mice. Nonetheless, Wnt7b overexpression modestly improved glucose tolerance in the male mice and seemed to suppress HFD-induced fat accumulation in both sexes. Thus, bone-specific Wnt7b overexpression appears to improve certain aspects of whole-body metabolism in association with enhanced bone anabolism in mice on HFD.

## Materials and methods

### Mouse strains and diets

All mouse procedures were approved by the Institutional Animal Use and Care Committee at Children’s Hospital of Philadelphia (IAC 21-001296). Mice were housed in groups with maximum of 5 adults per cage at 22°C with a 12:12-h light–dark cycle. The mouse strains of Osx-rtTA, tetO-Cre, or R26-Wnt7b are as previously described.^22^^,^^23^^,^^29^ All mouse strains are in C57BL6/J background. For doxycycline (Dox) treatment, mice were fed Dox food (Envigo, TD.04104) for 2 wk beginning at 4 wk of age. Subsequently, the mice were switched to a high-fat diet (HFD) (Research Diets Inc. D12492, 60 kcal% fat) or a regular chow diet (LabDiet, Mouse Diet 5015, 26 kcal% fat) for 23 wk before being sacrificed. Both males and females were studied and analyzed separately.

### DXA analyses

Animals were anesthetized with isoflurane before DXA at 29 wk of age before euthanasia. A Faxitron DXA X-ray cabinet was used to calculate lean and fat mass/percentage, as well as BMD)for both whole body and the right hindlimb (femur, patella, tibia, and fibula). Animals were measured blindly. Based on pilot results with Wnt7b overexpression causing large increases in BMD in the current setting, a sample size of 3 per group had a statistical power of 0.9 to detect the differences with alpha at 0.05.

### μCT analyses

The distal end of the femur was scanned using micro-computed tomography (μCT; μCT45, SCANCO Medical) at an isotropic voxel size of 4.5 μm. For trabecular bone analyses, ROIs were selected spanning 0.45–2.25 mm below the growth plate. For cortical bone analyses, a total of 70 slices at the femur midshaft, located approximately 5.4 mm from the distal growth plate, were analyzed. Parameters derived from these data included bone volume (BV), total volume (TV), and BV/TV ratio at the distal femoral metaphysis, as well as total area (TA), bone area (BA), and BA/TA ratio at the mid-diaphysis of the femur. Measurements were conducted blindly. A power analysis based on pilot data indicated a sample size of 3 per group had a statistical power of 0.9 to detect the large differences in trabecular or cortical bone mass between control and Wnt7b overexpression mice.

### GTT and ITT

Glucose tolerance test (GTT) and insulin tolerance test (ITT) were performed with 1-wk interval to allow for recovery from stress. The mice were fasted for 6 h before each test. For GTT, glucose was injected intraperitoneally at the dose of 2 g/kg body weight. For ITT, insulin was injected intraperitoneally at 0.5 U/kg body weight (Novalin R U-100). For both tests, glucose levels were monitored with a glucometer by collecting blood drops from tail cuts at the indicated time points.

### Serum biochemical assays

After a 6-h fasting period, serum was collected from mice via retro-orbital bleeding. Glucose levels were then measured using the Glucose (HK) Assay Kit (Sigma, GAHK20). C-terminal Telopeptides of Type I Collagen (CTX-I) and Procollagen 1 N-terminal Propeptide (P1NP) assays were performed using the RatLaps CTX-I ELISA and Rat/Mouse P1NP EIA Kit (Immunodiagnostic Systems, Ltd). Carboxylated and uncarboxylated osteocalcin levels were measured using the Mouse Gla-Osteocalcin High Sensitive EIA Kit (Takara, MK111) and Mouse Glu-Osteocalcin High Sensitive EIA Kit (Takara, MK118), respectively. The lipid metabolite assays were conducted at the University of Pennsylvania Rodent Metabolic Phenotyping Core as follows. Triglycerides was measured with Stanbio Triglyceride LiquiColor Test (Enzymatic) (Catalog # 2100-430). Cholesterol was measured with Stanbio LiquiColor Enzymatic Cholesterol Test (Catalog # SB-1010-225). Non-esterified fatty acid (NEFA) was measured with Fujifilm Wako HR Series NEFA-HR(2).

### Statistical analyses

Statistical significance was determined using either an unpaired Student’s *t*-test or 2-way ANOVA with the 2-stage step-up method of Benjamini, Krieger, and Yekutieli for multiple comparisons.

## Results

### Wnt7b overexpression in osteoblasts leads to massive bone accrual

To investigate the potential effect of Wnt7b overexpression in bone on whole body metabolism, we fed the experimental vs control mice with an HFD. To this end, we crossed Osx-rtTA;tetO-Cre double hemizygous mice with R26-Wnt7b homozygous mice to generate experimental mice with the genotype of Osx-rtTA;tetO-Cre;R26-Wnt7b, hereafter referred to as *o*ver*e*xpression or OE mice. We have previously shown that the OE mice activate Wnt7b expression from the Rosa26 locus permanently in osteoblasts upon Dox treatment.^23^ The littermate animals, carrying one allele of R26-Wnt7b but lacking either Osx-rtTA or tetO-Cre, were not expected to respond to Dox and served as the control group (Ctrl). To consider potential sex differences, male and female mice were analyzed separately.

**Figure 1:**
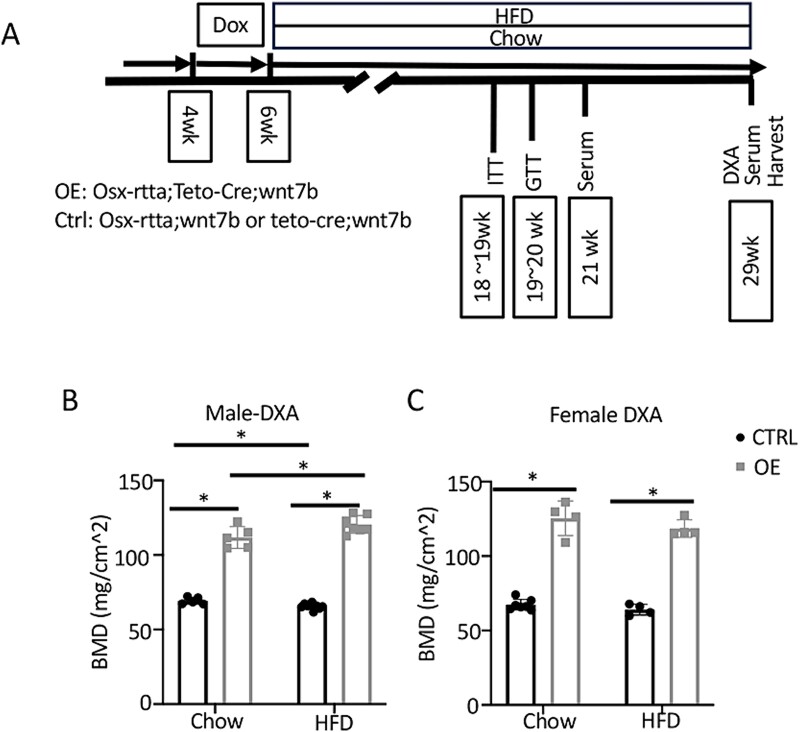
**Wnt7b overexpression protects against HFD-induced bone loss.** (A) Study design. Both males and females were included in the study and analyzed separately. (B, C) BMD analyses by DXA in 29-wk-old male (B) and female (C) mice. Abbreviations: Ctrl, Control; HFD, high fat diet; OE, Wnt7b overexpression. Each data point represents a single mouse. Data presented as mean ± SD. ^*^p<.05, 2-way ANOVA.

In the experiment, OE or Ctrl mice were fed with Dox food for 2 wk starting at 4 wk of age and then switched to either HFD or a standard chow diet for 23 wk before harvest (Figure 1A). Whole body BMD was measured with DXA at the time of harvest (DXA). In addition, ITT and GTT were performed at 12–13 wk and 13–14 wk, respectively, after Dox to monitor potential changes in insulin sensitivity and glucose handling (Figure 1A). Sera were collected both at 15 wk after Dox and at harvest for biochemical measurements. In keeping with our previous studies, Wnt7b OE mice markedly increased whole body BMD over the Ctrl in both male and female mice on regular chow diet^23^ (Figure 1B and C). Interestingly, HFD feeding for 23 wk slightly decreased in the BMD of male Ctrl mice, but the effect was completely reversed in the male OE mice, which in fact exhibited a slightly higher BMD than those on chow diet (Figure 1B). In contrast, HFD for the same duration did not change the BMD of either Ctrl or OE females (Figure 1C). Overall, bone-specific overexpression of Wnt7b greatly increased whole body mineral density in both male and female mice on either regular chow or HFD.

**Figure 2:**
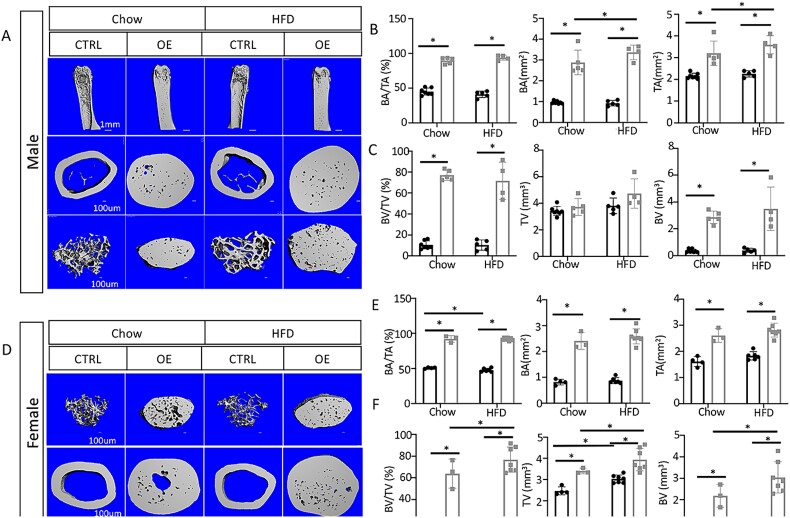
**Wnt7b overexpression in osteoblasts causes severe osteosclerosis.** (A and D) Representative μCT images of 3D reconstruction of distal femurs in males (A) or females (D). μCT quantification of cortical bone parameters in males (B) or females (E). BA, bone area; TA. total area. μCT quantification of trabecular bone parameters in males (C) or females (F). Abbreviations: BV, bone volume; TV: total volume. Each data point represents a single mouse. Data presented as mean ± SD. ^*^p<.05, 2-way ANOVA. μCT, micro-computed tomography.

We next performed μCT analysis on long bones harvested at the terminal timepoint to further characterize the high bone mass phenotype in OE mice. The images showed that entire skeletal elements were essentially filled with solid bone, with no clear separation of trabecular from cortical bone and with minimal marrow space, in all OE mice irrespective of sex or diet (Figure 2A and D). Quantification of the cortical bone in the males indicated that the cross-sectional TA and BA at the diaphysis were essentially equal in the OE and were both markedly greater than their counterparts in Ctrl mice, regardless of the diet (Figure 2B). Moreover, HFD further increased the diaphyseal bone (BA) and TAs specifically in the OE males (Figure 2B). Quantification of the metaphyseal trabecular bone confirmed a dramatic increase in BV but no change in tissue volume (TV), resulting in a nearly 8-fold increase in the fractional bone volume (BV/TV) in male OE vs Ctrl mice on either diet (Figure 2C). Likewise, the female OE mice exhibited notable increases in the diaphyseal or metaphyseal bone parameters when compared to Ctrl on either diet (Figure 2E and F). In addition, HFD slightly reduced the fractional bone area (BA/TA) of the diaphysis in the female Ctrl mice, but the effect was eliminated in the OE group (Figure 2E). Finally, HFD increased the metaphyseal tissue volume (TV) in both Ctrl and OE female mice but increased fractional bone volume (BV/TV) only in the female OE group due to increased BV (Figure 2F). Therefore, Wnt7b overexpression greatly increased bone mass in mice on either chow diet or HFD.

**Figure 3:**
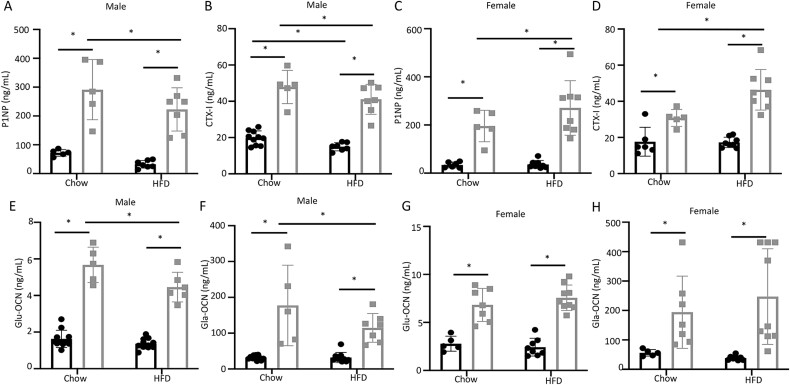
**Wnt7b overexpression increases bone formation and bone resorption markers and osteocalcin levels in the serum.** P1NP (A) and CTX-I levels (B) in male mice. P1NP (C) and CTX-I levels (D) in female mice. Glu-OCN (E) and Gla-OCN (F) levels in male mice. Glu-OCN (G) and Gla-OCN (H) levels in female mice. Each data point represents a single mouse. Data are expressed as mean ± SD. ^*^p<.05, 2-way ANOVA. Glu-OCN, uncarboxylated osteocalcin.

We next measured potential changes in bone formation or resorption activities. Specifically, sera collected shortly before euthanasia were used to measure the levels of bone formation marker P1NP and bone resorption marker CTX-I. Both P1NP and CTX-I levels were elevated in OE vs Ctrl mice, regardless of sex or diet (Figure 3A–D). Among the male mice, HFD appeared to suppress both P1NP and CTX-I levels in either Ctrl or OE mice, although the decrease in P1NP among the Ctrl did not reach statistical significance (Figure 3A and B). Contrary to the males, the OE but not Ctrl females increased both P1NP and CTX-I levels in response to HFD (Figure 3C and D). Thus, Wnt7b overexpression stimulates the overall activities of both bone formation and resorption, whereas HFD appears to affect bone turnover in a sex-dependent manner.

Osteocalcin is a predominant non-collagenous protein secreted by osteoblasts and its circulating levels can reflect changes in either bone formation or resorption. Whereas most osteocalcin in the serum is present in the carboxylated form (Gla-OCN) (carboxylated osteocalcin), a relatively minor fraction is undercarboxylated (Glu-OCN) and has been postulated to function as a hormone.^30^ We therefore quantified both Gla-OCN and Glu-OCN in the serum at the time of harvest. In both males and females and regardless of the diet, Glu-OCN and Gla-OCN levels were at least 3 times higher in the OE than Ctrl mice (Figure 3E–H). Interestingly, HFD reduced both Gla- and Glu-OCN selectively in the OE males (Figure 3E and F). The reductions were in keeping with the modest suppression of P1NP and CTX-1 levels by HFD in those mice (Figure 3A–B). Overall, consistent with its strong bone anabolic function, Wnt7b overexpression markedly elevated the circulating levels of OCN.

### Wnt7b overexpression reduces HFD-induced fat mass accrual

**Figure 4:**
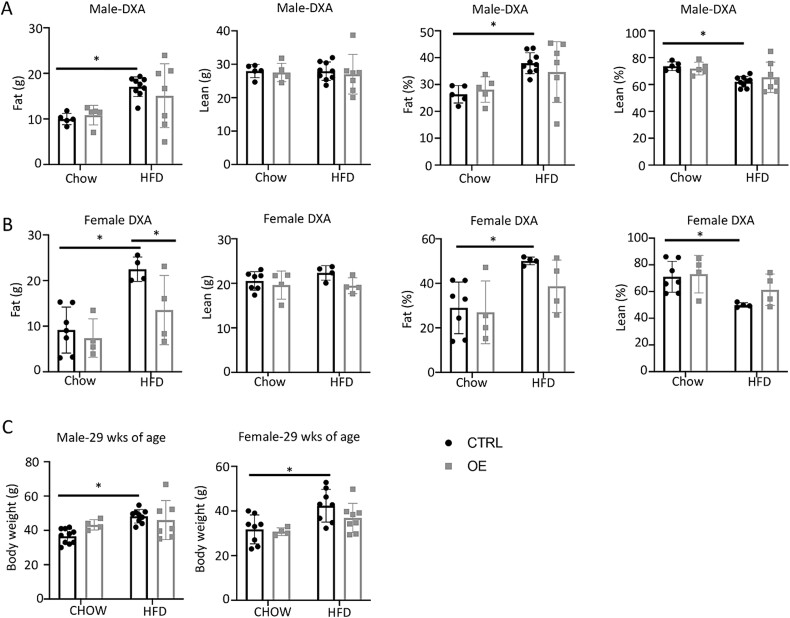
**Wnt7b overexpression reduces fat mass in mice on HFD.** Fat and lean mass and percentages determined by DXA in male (A) and female (B) mice at 29 wk of age. (C) Body weight measurements of males (left) and females (right). Each data point represents a single mouse. Data are expressed as mean ± SD. ^*^p<.05, 2-way ANOVA. Abbreviation: HFD, high-fat diet.

To assess the potential effect of Wnt7b overexpression on whole body metabolism, we performed body composition analyses with DXA at the time of harvest. As expected, the Ctrl mice of either sex gained fat mass after 23 wk of HFD feeding (Figure 4A and B). In contrast, neither male nor female OE mice significantly increased fat after HFD compared to chow diet (Figure 4A and B). However, due to the large individual variations, the relatively small number of animals was insufficiently powered to detect minor increases. Nonetheless, among the females on HFD, the OE mice possessed significantly less fat mass than Ctrl (Figure 4B). The lean mass remained stable between HFD and chow diet in both sexes of either genotype, resulting in a significant increase in fat percentage at the expense of lean mass specifically in the Ctrl but not OE mice on HFD (Figure 4A and B). Consistent with the body composition data, at the time of harvest, OE mice on either chow diet or HFD had a similar body weight even though the Ctrl mice increased body weight in response to HFD (Figure 4C). It is worth noting that the Ctrl and OE mice possessed similar total body weight despite more bone in the latter; this may indicate less fat mass in the OE mice than normal. Overall, the results support the notion that bone-specific Wnt7b overexpression suppresses fat accumulation induced by HFD in both sexes.

### Wnt7b overexpression affects glucose disposal in male mice

**Figure 5:**
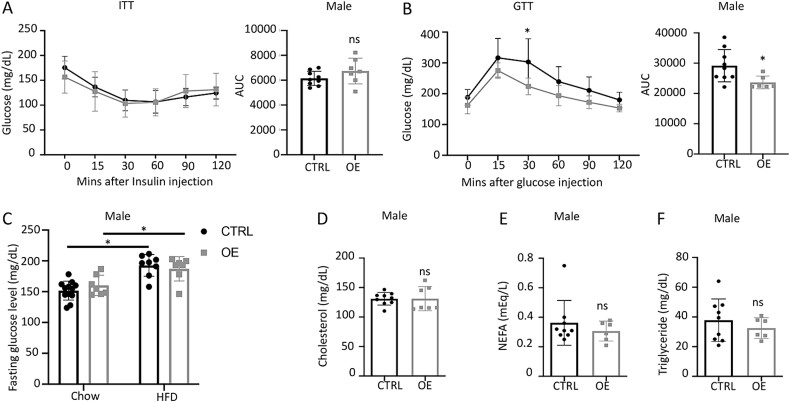
**Wnt7b overexpression in osteoblasts improves glucose tolerance but not insulin sensitivity in male mice on HFD.** (A) ITT (insulin tolerance test) at 18–19 wk of age. AUC, area under curve. (B) GTT (glucose tolerance test) at 19–20 wk of age. (C) Fasting serum glucose levels. (D–F) Fasting serum levels of cholesterol, non-esterified fatty acids (NEFA), and triglyceride at 21 wk of age. Each data point represents a single mouse. Data are expressed as mean ± SD. ^*^p<.05, ns, not significant, Student’s *t*-test (A, B, D–F) or 2-way ANOVA (C). Abbreviations: GTT, glucose tolerance test; HFD, high-fat diet; ITT, insulin tolerance test.

To examine potential changes in global glucose metabolism, we performed GTT and ITT among the HFD-fed mice at 12–13 wk after the initiation of HFD. Among the males, there was no difference in ITT between the genotypes, but GTT showed better performance in the OE group than Ctrl (Figure 5A and B). We further measured fasting glucose levels in the serum at harvest time. The fasting glucose levels increased in both Ctrl and OE male mice following HFD feeding compared to the chow diet, with no difference between the genotypes (Figure 5C). We also used the serum collected at 15 wk after HFD (21 wk of age) to evaluate lipid metabolism and detected no difference in cholesterol, NEFAs, or triglycerides between the HFD-fed Ctrl and OE mice (Figure 5D–F). Thus, bone-targeted overexpression of Wnt7b in the male mice appeared to improve global glucose tolerance without affecting insulin sensitivity or lipid metabolism after HFD feeding.

**Figure 6:**
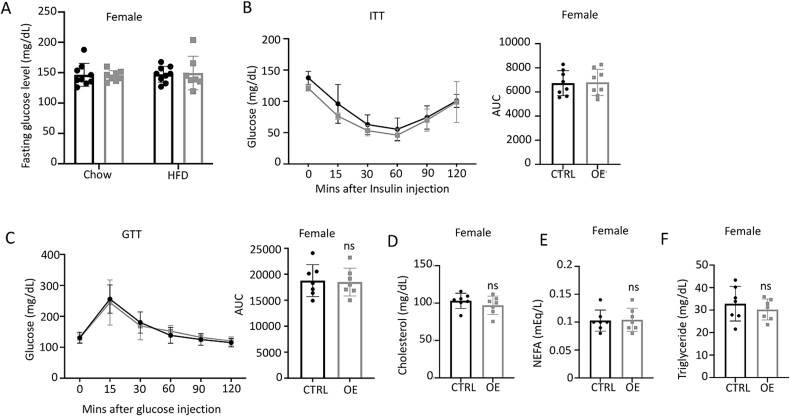
**Wnt7b overexpression in osteoblasts does not affect glucose tolerance or insulin sensitivity in female mice on HFD.** (A) Fasting serum glucose levels at 29 wk of age. (B) ITT at 18–19 wk of age. AUC, area under curve. (C) GTT at 19–20 wk of age. (D–F) Fasting serum levels of cholesterol, non-esterified fatty acids (NEFA), and triglyceride at 21 wk of age. Each data point represents a single mouse. Data are expressed as mean ± SD. Two-way ANOVA (A) or Student’s *t*-test (B–F). Abbreviations: GTT, glucose tolerance test; HFD, high-fat diet; ITT, insulin tolerance test.

In contrast to the males, the female Ctrl or OE mice maintained normal fasting glucose levels after HFD feeding (Figure 6A). The apparent metabolic resistance of female mice to HFD is consistent with previous reports and likely reflects the protective effect of estrogen.^31^ Moreover, GTT, ITT, or the lipid assays did not reveal any difference between the genotypes in response to HFD (Figure 6B–F). Therefore, even though Wnt7b overexpression in osteoblasts dramatically increased bone mass, it did not alter whole body glucose or lipid metabolism in female mice.

## Discussion

The study shows that bone-specific overexpression of Wnt7b markedly increased bone mass in mice even after prolonged feeding of HFD. Metabolically, Wnt7b overexpression appeared to reduce body fat gain caused by HFD in both sexes and modestly improved glucose tolerance specifically in the male mice, but it did not modify fasting glucose levels or insulin sensitivity in either sex. The results therefore demonstrate a strong bone anabolic activity of Wnt7b in association with an apparent reduction in fat gain in overnourished mice.

The Wnt7b-overexpressing mice exhibited a 3-fold increase in the circulating Glu-OCN, and therefore offered a useful model for assessing the potential pharmacological role of osteocalcin as a metabolic hormone. We performed the metabolic measurements in the mice on HFD as previous studies showed that mice on a similar diet and infused with recombinant osteocalcin notably improved metabolic parameters including fasting serum glucose and triglyceride levels, GTT, and ITT.^8^ However, we did not see any changes in ITT, fasting glucose, or triglyceride levels in the OE mice, even though a slight improvement of GTT was observed in the HFD-fed males. Because glucose homeostasis in the Ctrl female mice appeared to be impervious to HFD, a potential effect of increased Glu-OC on dysregulated glucose metabolism in females could not be assessed in the current setting. In any case, the apparent disagreement between the current and previous findings could potentially be explained by differences in the genetic increase vs osmotic pump delivery of osteocalcin. For example, the pump-delivery method could have led to a higher level of circulating osteocalcin than what was achieved in our genetic model, but serum osteocalcin levels were not determined in the previous study. The modest improvement in glucose tolerance in the male OE mice on HFD was in keeping with a potential positive effect of osteocalcin on glucose homeostasis, but such a role is difficult to untangle from the overall reduction of body fat in those mice. Altogether, our results do not confirm a strong role for increased uncarboxylated osteocalcin in improving glucose metabolism in mice on HFD.

It was somewhat unexpected that HFD, when compared to the regular chow, had little effect on the bone parameters as measured by μCT in the control mice. Previously we found that female but not male mice on the same HFD (60% fat kcal) reduced trabecular bone mass when compared to those fed a nutritionally matched low-fat diet (LFD).^32^ The LFD contained less fat (10% fat kcal) than the regular chow (25% fat kcal) used here. In addition, the previous study analyzed the bones at 11 wk of age, considerably younger than the 29 wk here. Therefore, a potential HFD effect could conceivably be masked by the trabecular bone loss associated with aging in the current study. Overall, the different outcomes between the studies underscore the sensitivity of bone mass to both diet compositions and age.

The mechanism for reduced fat mass in the Wnt7b-overexpressing mice is currently unclear. As Wnt proteins function predominantly in an autocrine or paracrine manner, Wnt7b-induced bone anabolism instead of Wnt7b per se was likely responsible for the apparent anti-obesity effect. However, it remains possible that osteoblast-derived Wnt7b enters the circulation to act directly on adipose tissues. Nonetheless, we envision that the increased bone mass could reduce fat accumulation through at least two potential mechanisms. First, bone may secrete certain yet unknown endocrine factors to suppress adipose tissues directly. In this regard, it is worth noting that osteocyte-derived Sost has been proposed to promote body fat formation via direct actions.^18^ Thus, a potential bone-derived anti-adipogenic factor would need to override the activity of circulating Sost, which may rise due to the increased number of osteocytes in the OE mice. Second, bone as an end user of energy substrates is expected to greatly increase nutrient consumption in the OE mice, and this could alleviate overnutrition caused by HFD, resulting in less fat deposition. In this regard, mice with hyperactive HIF signaling in osteoblast-lineage cells have been shown to increase glucose uptake in bone and improve global glucose metabolism.^33^ Future studies are warranted to distinguish those possibilities and to elucidate the exact mechanism in our mouse model.

It is worth reiterating that bone is a major end user of energy substrates in the context of global metabolism. Osteoblasts require a large energy supply to synthesize, deposit, and mineralize bone matrix during bone formation.^34^ Glucose was estimated to contribute ~80% of energy production in osteoblasts in vitro, whereas the skeleton accounted for approximately 15% of glucose uptake from the blood in healthy mice.^33^^,^^35^ Furthermore, increased glucose utilization by activation of HIF signaling in osteoblasts not only increased bone formation but also improved systemic glucose clearance.^33^^,^^36^ Besides glucose, both fatty acid and glutamine consumption have been shown to increase in osteoblasts in response to Wnt stimulation.^27^^,^^28^ Of note, osteosclerosis in the OE mice, as assessed by μCT, was further accentuated upon HFD feeding, supporting the view that increased nutrient supply accelerated bone overgrowth. Overall, the findings suggest that increased bone anabolism by Wnt7b may alleviate nutrient surplus in mice by boosting energy substrate consumption, but such a model remains to be tested directly in the future.

## Data Availability

Original data are available upon request.
